# Is long-term memory used in a visuo-spatial change-detection paradigm?

**DOI:** 10.3758/s13423-021-01951-8

**Published:** 2021-06-08

**Authors:** Benjamin Goecke, Klaus Oberauer

**Affiliations:** 1 Institute for Psychology and Pedagogy, Ulm University, Albert-Einstein-Allee 47, 89081 Ulm, Germany; 2 Department of Psychology, University of Zurich, Zurich, Switzerland

**Keywords:** Visual working memory, Long-term memory, Change-detection paradigm, Hebb repetition effect

## Abstract

In tests of working memory with verbal or spatial materials, repeating the same memory sets across trials leads to improved memory performance. This well-established “Hebb repetition effect” could not be shown for visual materials in previous research. The absence of the Hebb effect can be explained in two ways: Either persons fail to acquire a long-term memory representation of the repeated memory sets, or they acquire such long-term memory representations, but fail to use them during the working memory task. In two experiments (*N*_*1*_ = 18 and *N*_*2*_ = 30), we aimed to decide between these two possibilities by manipulating the long-term memory knowledge of some of the memory sets used in a change-detection task. Before the change-detection test, participants learned three arrays of colors to criterion. The subsequent change-detection test contained both previously learned and new color arrays. Change detection performance was better on previously learned compared with new arrays, showing that long-term memory is used in change detection.

*Repetitio est mater studiorum*—repetition is the mother of study. This fundamental principle probably applies to all entities that are able to do what we call “learning”; animals and humans, and even computers. Typically, learning requires the repetition of some target information, either intentionally or unintentionally. Across repetitions, neural connections in our brains gradually change to capture the repeated information.

What is the role of working memory for learning? Across 6 decades, several theorists have assumed that short-term or working memory (WM)—a medium for temporarily maintaining information—is the gateway into long-term memory (LTM), where the information is stored permanently. Atkinson and Shiffrin (1968) assumed that information must pass through the “short-term store” into LTM. Baddeley et al. (1998) hypothesized that the phonological loop, a component of Baddeley’s model of WM, is a device for learning new word forms. Recently, Cowan (2019) has emphasized that maintaining information in WM involves not only activating existing LTM representations, but also forming new ones. Forsberg et al. (2020) argued that the limited capacity of WM forms a bottleneck for the acquisition of new knowledge in LTM.

An important tool for studying the role of WM in the gradual acquisition of knowledge through repetition is the so-called *Hebb repetition effect* (Hebb, 1961). The Hebb repetition effect refers to the observation that immediate serial recall—a common test of WM—gradually improves for a memory list that is repeated several times over the course of an experiment (e.g., Hebb, 1961; Hitch et al., 2005; Page et al., 2006). The Hebb effect was originally observed in a task of immediate serial recall with verbal stimuli (Hebb, 1961), and is specifically discussed for its contribution to language learning (Lafond et al., 2010; Szmalec et al., 2009). Other studies found it also with meaningful visual stimuli like upright faces (Horton et al., 2008), and with sequences of spatial locations (e.g., Couture & Tremblay, 2006; Gagnon et al., 2004; Page et al., 2006; Turcotte et al., 2005).

In contrast, several attempts to demonstrate the Hebb effect with arrays of simple visual stimuli have largely failed. In particular, no improvement of change detection—a common test of visual working memory—has been found across dozens of repetitions of the same array (Fukuda & Vogel, 2019; Logie et al., 2009; Olson & Jiang, 2004). There is some evidence for learning with a change-detection paradigm (Shimi & Logie, 2019), but it appears to require many more repetitions (>60 in that study) than the classic Hebb effect, which is robust after about 10 repetitions.

## The present study

There are two possible explanations for the absence of Hebb learning in the change-detection task. First, participants could fail to acquire LTM representations about the repeated memory arrays. Second, participants might encode the repeatedly presented arrays in LTM, but fail to use these LTM representations in subsequent change-detection trials using the same arrays again. That is, although participants acquire knowledge with which they could improve their performance on repeated arrays, they do not. Some evidence for the latter possibility comes from two studies showing that, although change detection did not improve on repeated arrays, participants were able to recognize the repeated arrays well above chance in an end-of-experiment test (Fukuda & Vogel, 2019; Olson & Jiang, 2004).

In the current study, we aimed at testing whether long-term memory representations are used in a visuo-spatial change-detection paradigm. We created LTM traces of three six-color target arrays A, B, and C in a learning phase. We then compared the performance in a subsequent working memory test between trials using one of these target arrays, which demonstrably have been stored in LTM after the learning phase, and trials with randomly generated arrays (D) without representation in LTM. The experimental generation of LTM traces outside of the change-detection procedure allows for a distinction of two possible outcomes. If these representations are used, we should observe better change-detection accuracy for learned arrays as compared with random arrays. If these representations are not used, then the accuracy of the learned arrays should not be different from the accuracy of random arrays.

## Method

### Participants

Two different samples participated in Experiment 1 and Experiment 2, respectively. For Experiment 1, the sample consisted of *N* = 18 (*M*_age_ = 22.6 years, *SD*_age_ = 2.89) university students from the University of Zurich. Experiment 2 enrolled *N* = 30 (*M*_age_ = 23 years, *SD*_age_ = 4.89) university students of the University of Zurich and of Ulm University, of which one person was excluded from data analysis after the learning phase due to insufficient performance in the learning phase (final *N* = 29). Our choice of sample sizes was informed by the *N* of previous studies on the Hebb effect. We decided to increase the sample size of Experiment 2 to increase our chance of measuring a small effect of knowledge that we might have missed in Experiment 1. Both experiments were advertised via flyers and e-mail. Participants had to be between 18 and 35 years old and fluent in German. Interested persons were excluded from participation, if they were color-blind, or had poor (i.e., not corrected) eyesight.

### Materials and procedure

The tasks of both experiments were designed to study the same research question; however, some slight adjustments were made to the tasks after Experiment 1 was conducted. Both experiments consisted of two parts. The first was a learning phase, in which participants were instructed to learn three distinct color arrays (labelled A, B, and C, respectively; for the remainder of this paper they will be called “target arrays”). After that, a change-detection task was administered in which some trials used the target arrays, and other trials used new random arrays as memory sets.

#### Experiment 1

The learning phase of Experiment 1 started with the successive presentation of the three to-be-learned color arrays for 10 s each. Each array consisted of six color patches, which were distributed equidistantly on an imaginary circle, and to facilitate learning, each array was paired with a letter (A, B, or C, respectively) in the center of the imaginary circle. For all participants, the colors for each array were randomly chosen from a sample of 12 distinct colors (see Table 1 for RGB values).

**Table 1:** Colors and RGB values

Color	RGB value
Black	(0, 0, 0)
White	(255, 255, 255)
Blue	(0, 0, 255)
Red	(255, 0, 0)
Green	(0, 255, 0)
Yellow	(255, 255, 0)
Purple	(160, 32, 240)
Brown	(165, 42, 42)
Orange	(255, 165, 0)
Pink	(255, 192, 203)
Light-blue	(173, 216, 230)
Magenta	(255, 0, 255)

After this initial exposition to the three arrays, the learning phase was implemented by means of a classical change-detection paradigm. We decided to have participants learn the arrays in this way, because we wanted the learning experience to resemble the WM task. This procedure should ensure a low threshold for using the LTM representations during the subsequent WM test because it optimizes transfer-appropriate processing (Morris et al., 1977).

**Fig. 1:**
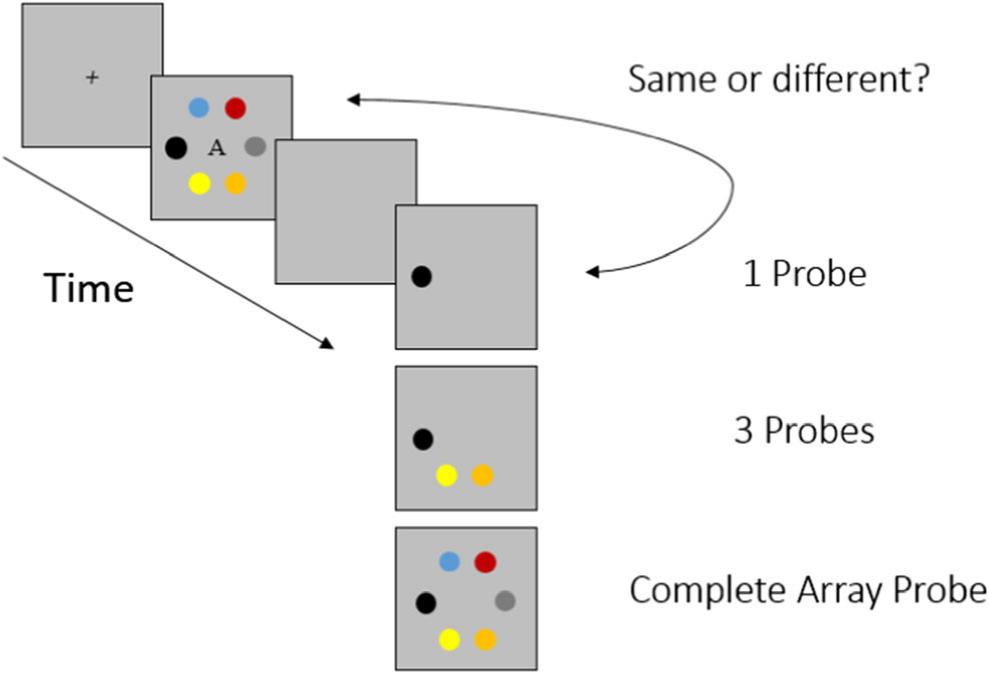
Schematic depiction of the change-detection paradigm of the learning phase in Experiment 1. (Color figure online)

The change-detection paradigm administered during the learning phase is schematically presented in Fig. 1. It consisted of three blocks with 24 trials each. Across all blocks, each trial started with the presentation of a fixation cross for 1,000 ms, which was centered on the screen. Next, one of the three target arrays was presented. Here, the presentation times varied between the three blocks. In the first block, the target arrays were presented for 3,500 ms, in the second block for 2,500 ms, and in the third block for 1,500 ms. After the presentation of a target array, participants were presented with a blank screen for another 1,000 ms. Subsequently, the participants were presented with a probe of the target array, consisting of one color patch in the first block, three color patches in the second block, or a complete array of six color patches in the third block. Participants had to indicate whether the now presented color patch(es) matched those of the complete array previously presented in the same position. Response time was not limited. There were three types of trials: no-change trials (same color patch in same position), swap-change trials (presented color patch in another position), and random-change trials (colors not previously presented in the target array in any position). Within each block, there were 12 no-change trials, six swap change trials, and six random change trials—the trial type order was randomized. Each array was presented eight times per block, and their order was randomized. After each trial, feedback was provided. If a response was correct, participants were presented with the message “Richtig!” (Correct!), and the next trial followed. If a response was incorrect, participants were presented with the message “Leider nicht richtig! So sieht die korrekte Anordnung aus:” (Unfortunately incorrect! This is what the correct array looked like:) and were then again presented with the complete target array to provide another learning opportunity. To ensure learning of the three target arrays, participants had to meet a learning criterion within each block, which was a minimum of 19 correctly answered trials (out of 24). If participants failed to meet this criterion, they had to repeat the block in which they failed to do so. In addition, at the beginning of each block the three target arrays were again presented for 10 s each. In this way, we hoped to induce a reasonably strong LTM representation of the target arrays.

Once a participant met all criteria of the learning phase, they were presented with the instructions of the subsequent working memory task—namely, another change-detection task. The instructions made the procedure of this task clear once again, and explicitly stated that no more feedback would be provided going forward. Generally, the change-detection paradigm strongly resembled the learning phase. Each trial started with the presentation of a fixation cross for 1,000 ms on a blank screen. Next, a six-color array was presented, but in contrast to the learning phase, now the presentation time was 1,000 ms. After a 1,000-ms retention interval, during which the screen was blank, one color patch was displayed as probe in the position of a randomly selected array item. Again, participants had to indicate whether the now presented color patch matched the color patch of the complete six-color array in the same position. Response time was not limited. For this working memory task, 10 blocks with 18 trials each were administered. Prior to that, participants had to complete 18 practice trials.

Of the overall 180 test trials, 90 trials presented one of the target arrays A, B, or C (30 trials each, now presented without their labels), and the other 90 trials were reserved for the presentation of new arrays (D), generated at random with the constraint that they must not be identical to one of the target arrays. Prior to the task, participants were not told that the previously learned arrays could be presented again. Across the 18 trials per block, there were approximately 40% no-change and 60% change trials.^1^ Again, the trial type order and the order of arrays was randomized within each block.

#### Experiment 2

**Fig. 2:**
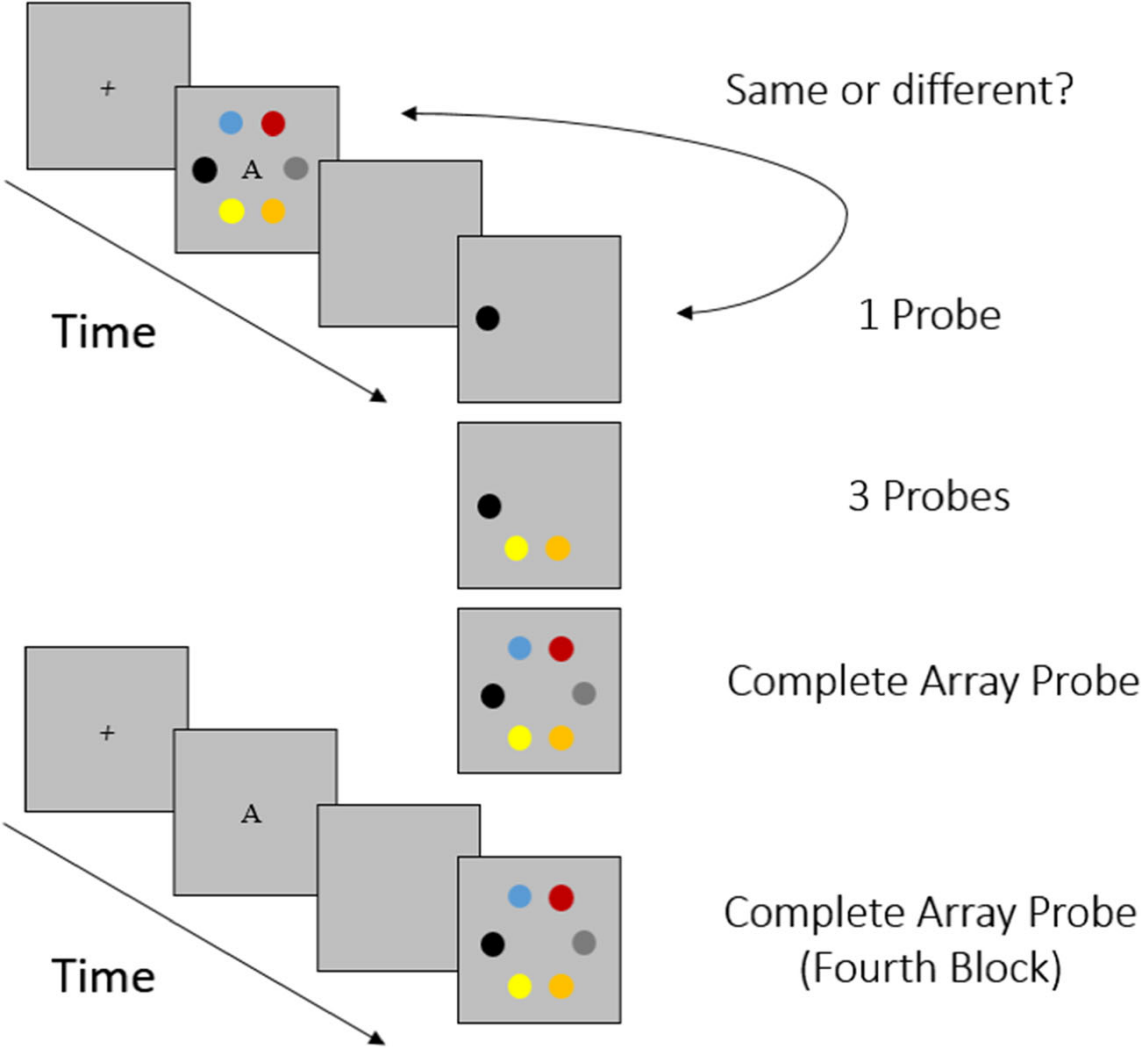
Schematic depiction of the change-detection paradigm of the learning phase in Experiment 2. (Color figure online)

Experiment 2 started with a similar learning phase as Experiment 1. However, to improve LTM learning, we added one more block of learning, and made the learning criterion for each block stricter (at least 20 out of 24 trials correct). For a schematic overview of the learning conditions, see Fig. 2. The learning phase now consisted of four blocks with 24 trials each. The first three blocks were the same as in Experiment 1, except that the presentation times for the target arrays across all blocks were randomized within a range between 1,000 and 5,000 ms. The fourth block added a new learning experience: Participants were now presented only with the labels A, B, or C, followed by a complete array probe. They had to indicate whether this probe matched the array they had learned to associate with the previously presented letter. This learning condition should ensure that participants had built LTM representations of the target arrays that they could retrieve on the basis of an arbitrary retrieval cue.

To test LTM after the learning phase, we added a discrete retrieval task, in which participants were presented with letter cues of the target arrays and six empty circles in the positions of the color patches. The six empty circles were marked one after another, and participants had to choose the correct color out of a set of 12 distinct colors, presented next to the empty array. The marked circle was then filled with the chosen color, if the choice was correct. If a choice was incorrect, participants were notified, and the actually correct color was filled in. This way, participants were once again presented with the complete target arrays and were able to further adjust their LTM representations of them.

The following WM phase was almost identical to Experiment 1. We reduced the presentation times of the arrays to 250 ms. In addition to that, the ratio of the probe types was changed due to a programming error from a ratio of 40:60 between no-change and change probes to approximately 70% no-change probes and approximately 30% change probes (separated into swap changes and random changes).

After the WM test, the participants were again presented with the discrete retrieval task, and a repetition of the new fourth block of the learning phase, with letters as cues for the target arrays, in order to test their LTM representations of the target arrays one last time. This allowed us to compare the accessibility of the target arrays in LTM both before and after the WM task.

The general procedure for both experiments was similar. Both test sessions lasted approximately 1.5–2 hours, and participants were compensated either with 15–22 CHF or partial course credit. Prior to participation, all participants provided informed consent. The experiments were supervised by trained research assistants. The tasks were programmed in and presented via PsychoPy 2 (Peirce et al., 2019). All tasks and standardized instructions were presented on computer screens with a Full HD resolution (1,920 × 1,080 pixels). All stimuli were presented on a grey background color, and participants used marked keys (- and <) on standard keyboards for responding to the tasks.

### Data analysis

All statistical analyses were conducted with R (R Core Team, 2020). The main analyses were conducted with the R packages brms (Bürkner, 2017). To make all analyses transparent and reproducible, we provide all material necessary to reproduce the main findings in an online repository (https://osf.io/ax763/).

For both experiments, both the learning phase, and the WM test, provide a dichotomous dependent variable indicating accuracy. Therefore, we analyzed the data for the WM tests with logistic regression models, in which the number of correct responses was predicted by the type of the presented arrays (i.e., learned target arrays vs. not-learned random array). In addition to the fixed effect of this predictor, the full model included a main effect of block, an interaction term of array type with block, a random effect of the subject (i.e., random intercept), as well as a term for the effect of blocks and array types nested within subjects (i.e., random slopes). After specifying the full model, we compared it to more parsimonious models to evaluate the evidence for each single effect by means of Bayes factors for model comparisons (Bürkner, 2017). The priors for the mixed-effects logistic regression models were Cauchy priors with a scale of 1/√2, obtained by adjusting the recommendations of Gelman et al. (2008) (for more details on the choice of scale for logistic regression models, please see Oberauer, 2019). The models were estimated with 100,000 samples, generated through three independent Markov chains, with 2,000 warm-up samples each (i.e., 98,000 post warm-up samples in total).

Because in both experiments the proportion of same and change trials was not balanced, participants could have developed response biases, which would distort the proportion-correct measure as an index of memory quality. Therefore, we also evaluated performance by two measurement models that separate memory quality from bias. A much-discussed divide between theories of visual WM is between those that assume a continuously varying strength or precision of memory representations (Ma et al., 2014; Oberauer & Lin, 2017), and those that assume a binary distinction between items that are remembered and others that are not (Adam et al., 2017; Zhang & Luck, 2008). To do both perspectives justice, we applied a signal-detection measurement model to measure memory quality on a continuous scale of discriminability, and a high-threshold model to measure the number of items remembered. Specifically, we computed *d'* (discriminability) and *c* (response criterion) from signal detection theory (based on Macmillan, 1993; Stanislaw & Todorov, 1999), where we corrected for extreme hit-rates and extreme false-alarm rates (i.e., 0 or 1; see Hautus, 1995). In addition, we computed *P*_*mem*_ (the probability that a participant had the tested item in memory) and *g* (guessing probability for a “change” response) from a high-threshold model (Model 4 from Cowan et al., 2013). All indices were computed for both learned and random array performance. Within each experiment, we predicted the respective indices by means of linear regression models with array type as predictor and a random effect of the subject (i.e., random intercept). As the indices were computed from data aggregating over all trials, we could not include block as a predictor in these analyses.

## Results

### Experiment 1

#### Learning phase

In Table 2, we report the accuracy for the different blocks of the learning phase. Seven persons had to repeat one of the learning blocks once. No person had to repeat the last learning block, indicating good learning. This is also shown by the decreasing proportion of errors from block to block.

**Table 2:** Mean % errors during the learning phase of Experiment 1, with standard errors

		% Error (*SE*)			
Learning phase	*N*	All arrays	Array A	Array B	Array C
1 Probe	18	16.9 (2.9)	17.4 (2.9)	17.4 (3.1)	16.0 (2.8)
1 Probe repetition	3	13.9 (3)	14.6 (3.7)	6.3 (1.5)	20.8 (3.4)
3 Probes	18	13.2 (3.7)	16.7 (3.9)	13.9 (4.3)	9.0 (2.8)
3 Probes repetition	4	10.6 (1.4)	10.9 (.7)	11.9 (.3)	9.1 (2.5)
Complete array probe	18	3.2 (1.5)	3.5 (1.4)	2.8 (1.6)	3.5 (1.7)
Complete array probe repetition	0	–	–	–	–

*Note. N* reflects the number of participants who worked on a respective block; some participants did not reach the criterion of at least 19 trials correct in the first and in the second block (i.e., 1 probe condition, and 3 probe condition, respectively), and therefore had to repeat these blocks.

#### Working memory task

**Fig. 3:**
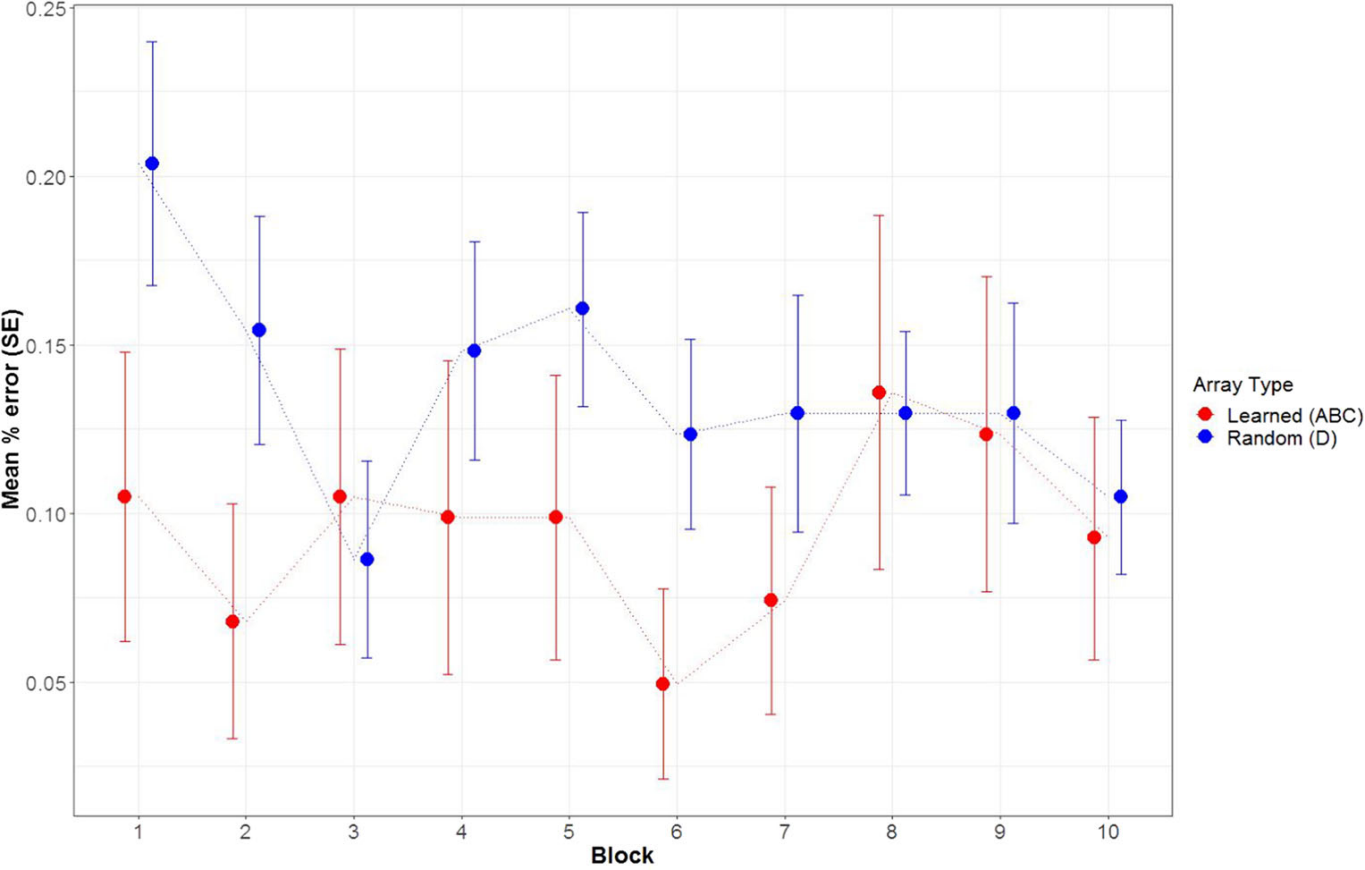
Mean performance of target and random arrays across 10 blocks in working memory task of Experiment 1. *Note*. Standard errors are depicted with error bars

Next, we present accuracy across the blocks of the working memory task (see Fig. 3). The performance on target arrays was better than on random arrays across most blocks. Furthermore, we did not observe a steady increase of performance across blocks specifically on the target arrays due to their repeated presentations across blocks, as would be expected if participants continued to learn these arrays during the change-detection task.

For the learned arrays, participants made 9.5% errors on average across all blocks, whereas they made 13.7% errors on average for the random arrays. This equals a standardized effect size of *d* = −.50 with a broad 95% CI [−1.05, .05].

The Bayes factors corresponding to the model comparisons for logistic regression models with and without specific effects are presented in Table 3. Only the main effect of array type was supported by this analysis, meaning that participants overall showed a better performance on learned arrays (corresponding parameter estimates can be found in Table 4).

**Table 3:** Bayes factors for single effects

Effect	*BF*
Random slopes (block and array type)	<.00001
Interaction (Array Type × Block)	.23
Main effect (array type)	132
Main effect (block)	.03

*Note.* BF = Bayes factor. The Bayes factors reflect the evidence for the model including a specific effect. The model containing a specific effect was always compared with the same model after excluding the corresponding effect. The effects were tested in the presented order, and all effects not supported were removed from both models in subsequent model comparisons.

**Table 4:** Parameter estimates of the best fitting model, including the main effect of array type

	*M*	*SD*	95% CI
Random effects			
*SD*_Intercept_	.66	.15	[.43, 1]
Fixed effects			
Intercept	1.96	.18	[1.62, 2.32]
Array type (learned vs. not learned)	.41	.11	[.19, .64]

### Experiment 2

#### Learning phase

In Table 5, we report descriptive statistics of performance in the learning phase. Twenty-two participants had to repeat at least one of the learning phases, as they did not reach the adjusted criterion of at least 20 trials correct. The number of repetitions for the one-probe condition ranged from 1 to 6, whereas the number of repetitions for the three-probe condition ranged from 1 to 4. However, we observed a clear trend of improvement across the learning blocks, indicating successful learning. No participant had to repeat the final two learning blocks.

**Table 5:** Mean % errors, with standard errors, of the target arrays during the learning phase of Experiment 2

		% error (*SE*)			
Learning phase	*N*	All arrays	Array A	Array B	Array C
1 Probe	30	18.6 (2.4)	19.6 (2.6)	21.2 (1.8)	15 (2.6)
1 Probe repetition	12	13.5 (1.7)	14.4 (1.4)	13.4 (1.8)	12.6 (1.9)
3 Probes	30	20 (2.7)	18.3 (2.7)	21.2 (2.5)	20.4 (2.8)
3 Probes repetition	15	11.8 (2.1)	9.17 (2)	13.3 (2)	13.1 (2.2)
Complete array probe	30	6.25 (1.5)	4.58 (1.4)	7.5 (1.5)	6.67 1.6)
Complete array probe repetition	0	–	–	–	–
Letter cue	30	8.3 (1.8)	6.25 (1.6)	10 (1.8)	8.75 (2)
Letter cue repetition	0	–	–	–	–

*Note. N* reflects the number of participants who worked on a respective block; some participants did not reach the criterion of at least 20 trials correct in the first and in the second block (i.e., 1 probe condition, and 3 probe condition, respectively) and therefore had to repeat these blocks.

Immediately following the learning phase, participants had to reproduce the previously learned arrays by manually picking out colors for each position of an array. For overall 18 to be filled out color patches (six per target array), participants had a mean correct of 67% (*SD* = 47%). This shows that participants were able to transfer their knowledge about the target arrays into another mode of retrieval (from change detection to recall). This discrete retrieval task was repeated after the WM test. Compared with the first retrieval task directly following the learning phase, the performance of the participants improved. For this last discrete retrieval task, 93% of the color patches were reproduced correctly on average (*SD =* 44.3%). A Bayesian *t* test for paired samples regarding the number of correct choices revealed weak evidence for better performance in the second discrete retrieval task compared with the first (*BF* = 3.59). This result shows that LTM traces of the target arrays did not decline throughout the WM test.

Finally, participants’ memory about the arrays was assessed one last time in the very end of the experiment by repeating the last block of the learning phase, where they only were presented with letter cues in a change-detection paradigm. They answered 93% of 24 trials correctly, indicating highly accurate and accessible knowledge of the target arrays.

#### Working memory task

**Fig. 4:**
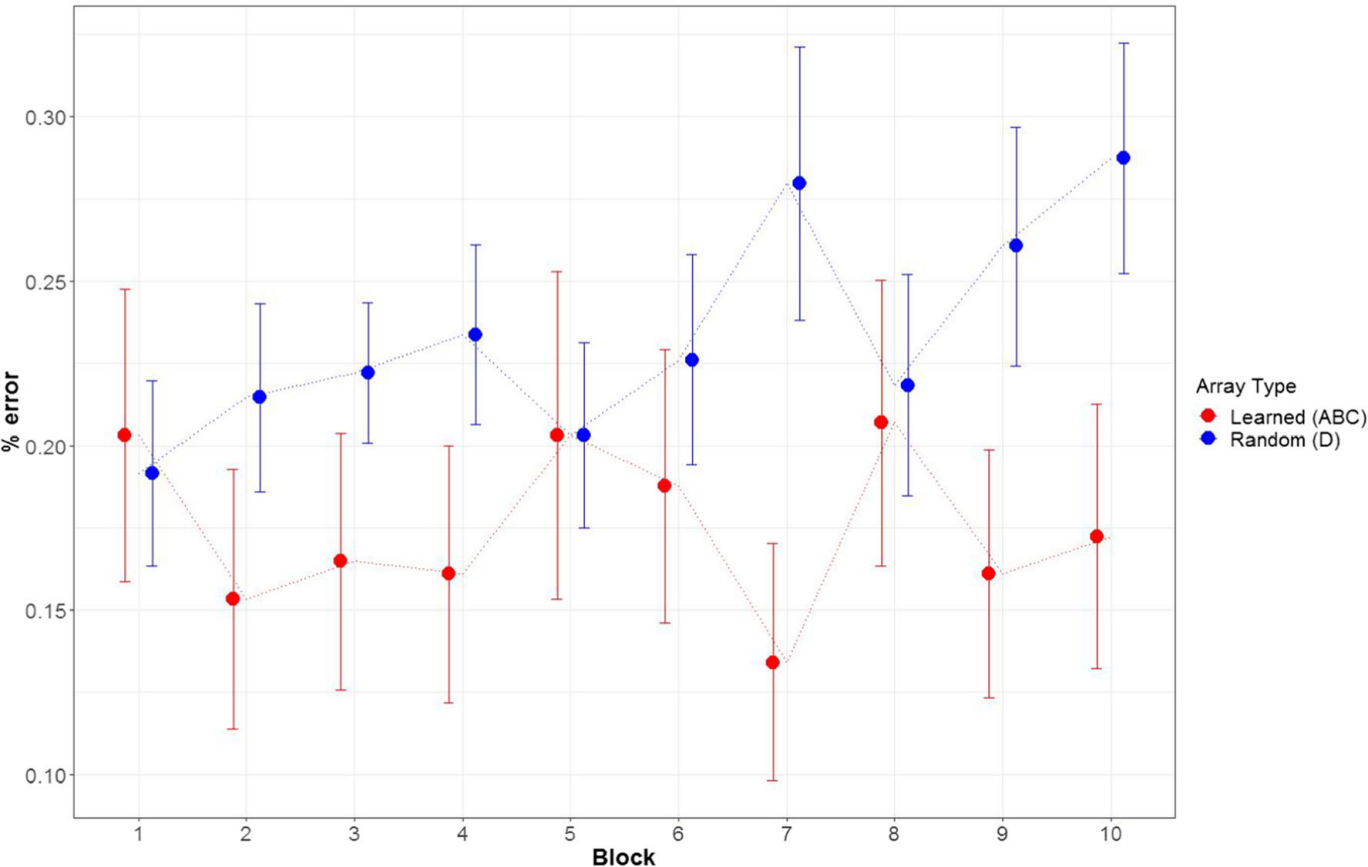
Mean performance of target and random arrays across 10 blocks in working memory task of Experiment 2. *Note.* Standard errors are depicted with the error bars

In Fig. 4, we present performance across the blocks of the change-detection task. The combined mean performance of the three arrays was better than for the random arrays in the majority of the blocks. On average, participants made 17.5% errors for the learned arrays, whereas they made 23.4% errors on average for the random arrays. This equals a standardized effect size of *d* = −.55 with a 95% CI [−.98, −.11]. Compared with the effect size of Experiment 1, the standardized mean difference in Experiment 2 is slightly larger. In addition, there was a tendency for the advantage of target arrays to increase across blocks, resembling a Hebb effect.

Please find the Bayes factors for the model comparisons for the logistic regression models in Table 6. We found decisive evidence for a main effect of array type. There was moderate evidence against the main effect of block, and against the interaction between both predictors. The final model thus included the main effect of array type and the random intercept (i.e., random effect of subject), and the corresponding parameter estimates can be found in Table 7.

**Table 6:** Bayes factors for single effects

Effect	*BF*
Random slope (block)	<.00001
Interaction (Array Type × Block)	.25
Main effect (array type)	> 10000
Main effect (block)	.09

*Note.* BF = Bayes factor. The Bayes factors present the evidence for a model, including an effect. The model containing a specific effect was always compared with the same model after excluding the corresponding effect. The effects were tested in the presented order, and all effects not supported were removed from both models in subsequent model comparisons.

**Table 7:** Parameter estimates of the best fitting model, including the main effect of array type

	*M*	*SD*	[95% Cred. Interval]
Random effects			
*SD*_Intercept_	.56	.09	[.41, .76]
Fixed effects			
Intercept	1.26	.11	[1.03, 1.49]
Array type (learned vs. not learned)	.38	.07	[.24, .51]

### Separating memory quality from response bias

In Table 8, we present the above-mentioned change-detection measurement model indices for each experiment, separated by array type. In addition to the descriptive statistics, we report the corresponding effect sizes for each index per experiment, and the Bayes factors reflecting the evidence for a main effect of array type.

**Table 8:** Descriptive and test statistics of measurement model indices for signal detection parameters, high-threshold parameters, and mean proportion errors per experiment and condition

Index	Array Type	Experiment 1	Experiment 2
		*M* (*SD*)	*M* (*SD*)
*d'*	not learned	2.24 (.55)	2.00 (.58)
*d'*	learned	2.75 (.79)	2.22 (.71)
Cohen’s *d*	learned vs. not learned	.75	.35
*BF*		239.3	.30
*c*	not learned	.48 (.21)	.44 (.38)
*c*	learned	.37 (.21)	.27 (.27)
Cohen’s *d*	learned vs. not learned	−.55	−.51
*BF*		8.43	476.5
*P*_*mem*_	not learned	.66 (.14)	.59 (.16)
*P*_*mem*_	learned	.76 (.16)	.68 (.14)
Cohen’s *d*	learned vs. not learned	.67	.53
*BF*		654	136
*g*	not learned	.81 (.10)	.74 (.19)
*g*	learned	.76 (.13)	.67 (.16)
Cohen’s *d*	learned vs. not learned	−.40	−.41
*BF*		2.3	87.4
% error	not learned	.137 (.07)	.234 (.11)
% error	learned	.095 (.09)	.175 (.11)
Cohen’s *d*	learned vs. not learned	−.50	−.55
*BF*		>10000	>10000

In Experiment 1, participants’ discriminability index *d'* was larger on learned arrays than on random arrays; in Experiment 2, there was no evidence to support that difference. The response criteria (*c*) in both experiments were comparable, and indicate a small bias towards reporting a change in the trials. As the response criteria for both experiments were largely of the same magnitude, the different ratios of same to change trials in the two experiments had little effect on participants’ behavior. We found evidence in both experiments that the response criteria were decreased for learned arrays, meaning that the tendency to indicate a change was considerably smaller for arrays with LTM representations.

Turning to the high-threshold measurement model, the probability of having the tested item in memory, *P*_*mem*_*,* was higher for learned than for not-learned arrays in both experiments. Likewise, the guessing probability (*g*) to guess “change” was decreased for learned arrays relative to not-learned arrays. This was especially the case for Experiment 2, whereas the evidence in Experiment 1 was ambiguous. Taken together, both measurement models converged on the conclusion that learned arrays differed from not-learned arrays in both memory quality and bias. When bias was accounted for, the *d'* index no longer showed a credible effect of learning in Experiment 2. In Experiment 1, both *d'* and *P*_*mem*_ showed a credible effect of learning. Please note that the observed differences in all measurement model indices between experiments were not substantial, as indicated by Bayesian *t* tests for unpaired samples (the corresponding *BFs* ranged from .32 to 2.8).

## Discussion

With two experiments, we investigated whether information about visual arrays stored in long-term memory was helpful for subsequent performance in a change-detection task using these arrays. We induced long-term memory representations prior to a change-detection paradigm and ensured that it was robustly learned. In addition, the memorized arrays were further repeated over the course of the working memory task, allowing for further learning. If knowledge of the target arrays acquired in the learning phase was used in the working-memory test, performance on the learned arrays should be better than on random arrays. In addition, if people continued learning about the target arrays through their repetition in the working-memory test, then their change-detection performance would steadily improve over the course of array repetitions.

Taken together, the findings of both experiments showed clear evidence for the assumption that already existing LTM representations of visuo-spatial stimuli (i.e., color arrays) are beneficial for working memory performance during a change-detection paradigm. In both experiments we identified a main effect of array type. Change detection performance was better on previously learned compared with new arrays, showing that long-term memory is used in change detection. There was no evidence for further learning during the working-memory phase in both experiments.

Why did most previous studies show no evidence of learning in change detection tasks? Our experiments rule out one explanation, which is that people learn the repeated arrays, but do not use their knowledge for change-detection decisions. This leaves the alternative that people do not learn the repeated arrays, or at least do not learn them sufficiently well. There are reasons to believe that some cumulative learning of repeated arrays does occur. One is that Shimi and Logie (2019) found a gradual improvement of change detection over 60 or more repetitions of the same array. Additional evidence comes from the studies by Olson and Jiang (2004) and Fukuda and Vogel (2019). Although both studies found no clear evidence that performance on repeated arrays during a change-detection task was superior as compared with random arrays, the participants of both studies were able to identify the repeated arrays during a follow-up recognition test at above-chance level. This means that at least some learning for the repeated information must have happened during the experiments, but apparently not enough to make that knowledge helpful for change detection.

This could be because in the final recognition tests of those earlier studies, participants had to discriminate repeated arrays from randomly composed new arrays, from which they differed in several items, whereas the change probes of the change-detection task differed from the presented arrays in only one item. People might have acquired partial knowledge of the repeated array—for instance, knowledge about pairs or triplets of colors—which is sufficient to discriminate them from entirely novel arrays, but rarely helps detecting a single change. Another possibility is that the knowledge acquired about repeated arrays is weak, so that it is slow to retrieve. In the change-detection test, there might be a race between retrieval of the just-presented array from WM, and retrieval of a matching trace from LTM. If retrieval from LTM is much slower than retrieval from WM, it would rarely win the race. By contrast, in the final recognition test, only LTM is available, and therefore people are likely to take their time to retrieve and use it. Either way, the LTM representations that are built gradually from experiencing repeated arrays during change detection accumulate very slowly—much slower than in typical Hebb repetition experiments—and therefore do not benefit change-detection performance unless the number of repetitions exceeds about 50. In contrast, knowledge acquired in a separate learning phase, as in our experiments, is strong enough to be useful in change detection from the start.

The poor rate of learning stands in contrast to the fairly rapid learning observed in the Hebb repetition paradigm with other kinds of materials (verbal items, spatial locations, faces) and other testing procedures (i.e., serial recall or reconstruction). Therefore, maintaining a set of items in WM is not enough to foster rapid learning. Something else about the information to be learned, or the procedure of testing WM, must influence the rate of learning. One possibility raised by Logie et al. (2009) is that in change detection, the change probes interfere with the long-term memory representation of repeated arrays, thereby slowing learning. Another possibility is suggested by a still unpublished series of experiments by Souza and Oberauer (2021): Robust Hebb learning of visual arrays was observed only if all array items were tested on each trial. It could be that LTM is built primarily when we retrieve information from WM or LTM (Sutterer & Awh, 2016), and hence, learning during change detection is slow, because each trial involves only a single test.

## Conclusion

When strong and comprehensive knowledge about visual arrays is available in LTM, it is used in a change-detection task. The absence of a typical Hebb repetition effect with visual arrays (Fukuda & Vogel, 2019; Logie et al., 2009; Olson & Jiang, 2004) is best explained by people failing to learn the complete arrays strongly enough over a limited number of repetitions.

## Footnotes

1 Due to a programming error, we deviated from the initially intended ratio of 50:50 between no-change and change trials.
